# Associations between individual characteristics, decision-making autonomy, and satisfaction in a continuing education program for adult women in Chile

**DOI:** 10.3389/fsoc.2026.1893170

**Published:** 2026-09-01

**Authors:** David Álvarez-Maldonado, Javier Labbé Cid, Nicolás Pablo Barrientos Oradini, Carmen Pénnanen Arias, Luis Felipe Vergara, Víctor Manuel Yáñez Jara, Rafael Romero-Meza, Juan Carlos Armijos

**Affiliations:** 1 Universidad Tecnológica Metropolitana, Santiago, Chile; 2 Instituto de Salud Pública, Universidad Andrés Bello, Santiago, Chile; 3 Faculty of Economics and Business, Universidad Alberto Hurtado, Santiago, Chile; 4 School of Business and Administration, Universidad Miguel de Cervantes, Santiago, Chile; 5 Investigadora independiente, Santiago, Chile; 6 Faculty of Business and Economy, Universidad Andrés Bello, Santiago, Chile; 7 Academic and Administrative Area, Institute of Public Health, Universidad Andrés Bello, Santiago, Chile; 8 Universidad Alberto Hurtado, Santiago, Chile; 9 Facultad de Economía y Negocios, Universidad Santo Tomás, Santiago, Región Metropolitana, Chile; 10 School of Business and Administration, Faculty of Engineering, Science and Technology, Bernardo O'Higgins University, Santiago, Chile

**Keywords:** academic satisfaction, adult education, autonomy, multiple regression, sociodemographic factors

## Abstract

**Introduction:**

Continuing education programs play an important role in promoting women's employability and social inclusion. However, satisfaction with these programs is influenced not only by participants' individual characteristics but also by broader social contexts, including family responsibilities, decision-making autonomy, and access to educational opportunities. This study examined the associations between sociodemographic characteristics, different forms of decision-making autonomy, and overall satisfaction with the Diploma in Digital Skills for Employment, implemented by Universidad Alberto Hurtado within the framework of the project Transfer, Training, and Promotion of Women's Employment in Urbanized Areas of the Metropolitan Region of Santiago, funded by the Regional Government of Santiago, Chile.

**Methods:**

A quantitative, correlational, and cross-sectional design was employed. The study included 1,794 adult women who completed a structured questionnaire at the end of the diploma program. Descriptive statistics, Pearson correlations, and multiple linear regression analyses were conducted. Internal consistency of the satisfaction scale was assessed using Cronbach's alpha and McDonald's omega, and the assumptions underlying the regression models were verified.

**Results:**

Participants reported very high levels of overall satisfaction with the diploma program (M = 4.44; SD = 0.71). The satisfaction scale demonstrated excellent internal consistency (Cronbach's α = 0.864; McDonald's ω = 0.868). The regression model explained a small proportion of the variance in satisfaction (*R*^2^ = 0.035), although educational attainment, number of children, household headship, and autonomy in decisions regarding the use of family income were significantly associated with satisfaction, all with small effect sizes.

**Discussion:**

The findings indicate that individual characteristics and autonomy-related dimensions account for only a limited proportion of women's satisfaction with the program. Rather than suggesting that gender inequalities have been overcome or that participants' broader social conditions have changed, the high levels of satisfaction appear to reflect a positive evaluation of the educational experience within their specific personal and social contexts. These results suggest that program-related factors—such as pedagogical quality, institutional support, perceived relevance, and alignment with participants' expectations—are likely to play a more influential role in shaping satisfaction than individual sociodemographic characteristics alone. Future research should incorporate contextual, motivational, and institutional variables to develop more comprehensive explanatory models of satisfaction in continuing education programs for adult women.

## 1 Introduction

Women's economic empowerment has become a central priority within international development agendas because of its contribution to gender equality, poverty reduction, and inclusive economic growth. Beyond increasing women's participation in the labor market, empowerment involves strengthening women's capacity to make autonomous decisions regarding their economic, social, and personal lives. According to UN Women (2022), empowerment encompasses interrelated dimensions of participation, autonomy, and social transformation, all of which depend on expanding women's access to education, employment opportunities, and lifelong learning. Consequently, continuing education has increasingly been recognized as a strategic policy instrument for promoting women's economic inclusion and reducing structural inequalities.

Continuing education programs provide opportunities for adult women to strengthen professional competencies, develop digital skills, improve employability, and facilitate labor market reintegration after periods of economic inactivity or caregiving responsibilities (Radovan and Makovec, 2015). In addition, lifelong learning contributes to the development of self-efficacy, sustained participation in adult education, and the acquisition of digital competencies that support labor market inclusion (Bandura, 2018; Boeren and Nicaise, 2013; Li and Xue, 2023). Across Latin America, these initiatives have gained particular relevance as governments and higher education institutions have implemented training programs aimed at improving women's employment opportunities while fostering greater economic autonomy and social inclusion. Their growing importance has also increased the need to evaluate not only educational outcomes but also participants' perceptions of the learning experience.

Within this context, student satisfaction has become one of the most widely used indicators of educational quality and program effectiveness. Satisfaction reflects participants' overall evaluation of their learning experience and has consistently been associated with academic engagement, persistence, completion, institutional reputation, and willingness to recommend educational opportunities to others (Tinto, 1975; Parasuraman et al., 1988; Elliott and Shin, 2002; Syahmer et al., 2022). In continuing education, where adult learners frequently balance study with employment, caregiving responsibilities, and other competing demands, understanding satisfaction is particularly important because positive educational experiences contribute to sustained participation in lifelong learning.

Most previous research has explained student satisfaction primarily through institutional and pedagogical factors. Teaching quality, curriculum design, learning resources, administrative services, institutional support, and the overall learning environment consistently emerge as the strongest factors associated with educational satisfaction (Douglas et al., 2008; Tsinidou et al., 2010; Dhawan, 2022). These findings suggest that satisfaction is fundamentally shaped by characteristics of the educational experience itself rather than by participants' personal backgrounds.

Nevertheless, learners do not enter educational programs under identical conditions. Adult women participating in continuing education differ substantially in their educational trajectories, family responsibilities, household roles, and levels of decision-making autonomy. These individual circumstances may be associated with expectations, perceived usefulness of training, and ultimately the way participants evaluate their educational experience. Previous research has shown that educational attainment, caregiving responsibilities, and women's autonomy are associated with educational participation, labor market outcomes, and economic empowerment (Boeren, 2016; Boateng et al., 2022). However, considerably less evidence is available regarding whether these individual characteristics are also associated with satisfaction once women participate in continuing education programs.

This gap is particularly relevant because many publicly funded training initiatives targeting women are designed around assumptions regarding participants' socioeconomic profiles and empowerment needs. If satisfaction varies according to individual characteristics, educational interventions could be adapted to better address the needs of different participant groups. Conversely, if these characteristics explain only a limited proportion of satisfaction, greater attention should be directed toward improving pedagogical quality, institutional support, and other program-related factors that may exert stronger influence on participants' educational experiences.

Against this background, the present study examines the association between selected individual characteristics—including educational attainment, number of children, household headship, and different dimensions of decision-making autonomy—and overall satisfaction among women enrolled in a university continuing education diploma program designed to strengthen employability and economic autonomy in Chile. Rather than assuming that these characteristics are the principal predictors of satisfaction, the study evaluates the extent to which they contribute to explaining variation in participants' evaluations of the program. In doing so, it provides empirical evidence that informs both the design of continuing education initiatives for adult women and the broader discussion regarding the relative contribution of individual and program-related factors in shaping educational satisfaction.

## 2 Theoretical and sociological framework

The conceptualization of student satisfaction within the field of adult and continuing education requires a multidimensional approach that transcends simple evaluative metrics. Satisfaction is not merely a reflection of a completed task but a complex sociological and psychological construct that mediates the relationship between the learner's expectations, their social reality, and the institutional offering. In this study, the framework is built upon three pillars: the sociological role of academic satisfaction, the cognitive process of expectation-disconfirmation, and the andragogical principles of adult learning within a gendered social context.

### 2.1 Academic satisfaction as a sociological proxy for quality

Within the sociology of education, student satisfaction has evolved from being viewed as a customer-service metric to a fundamental indicator of educational quality and program effectiveness. Satisfaction reflects a participant's global evaluation of their learning experience, serving as a proxy for institutional success. According to Tinto (1975), academic satisfaction is intrinsically linked to academic and social integration; when students feel that the program aligns with their values and goals, their level of engagement and persistence increases.

In the context of continuing education for adult women, satisfaction is particularly critical because these learners often face “time poverty” due to caregiving and household responsibilities. Therefore, their evaluation of a program is not just about the content but about the perceived “return on investment” of their limited time. Elliott and Shin (2002) argue that student satisfaction is an alternative approach to assessing quality that captures the subjective reality of the learner, which objective metrics like grades or completion rates might miss. This study adopts this perspective, viewing satisfaction as a multidimensional construct that includes the fulfillment of expectations, the achievement of learning objectives, and the willingness to recommend the experience to others.

### 2.2 Expectation-Disconfirmation Theory (EDT): a cognitive-sociological model

A central theoretical anchor of this research is the Expectation-Disconfirmation Theory (EDT), originally developed by Oliver (1980). EDT posits that satisfaction is the result of a comparison between prior expectations and perceived performance. When the perceived performance meets or exceeds expectations, “positive disconfirmation” occurs, leading to high satisfaction. Conversely, if the performance falls short of the expected standard, “negative disconfirmation” ensues, resulting in dissatisfaction.

This model is crucial for interpreting why women with higher educational attainment in our sample reported lower levels of satisfaction. Sociologically, individuals with more advanced prior education possess more sophisticated “evaluative criteria” and higher initial benchmarks for quality. Following the logic of Bhattacherjee (2001), prior experience acts as a lens that shapes how new educational services are perceived. For highly educated women, a program designed for a general population might result in a “ceiling effect” where the perceived added value is lower than expected, triggering a negative disconfirmation process. Thus, satisfaction is not just an absolute measure of quality, but a relative measure of how well a program bridges the gap between a participant's starting point and their desired outcome.

### 2.3 Andragogy and instrumental motivations in adult learning

The educational experience of adult women must be understood through the lens of andragogy, as defined by Knowles (1973, 1978). Unlike pedagogy, which is often teacher-centered, andragogy emphasizes that adult learners are self-directed and motivated by the immediate application of knowledge to their real-life problems. Merriam and Bierema (2013) highlight that adult education is often “instrumental”; it is sought to fulfill specific social roles or to navigate life transitions.

In this study, the positive (though small) association between the number of children and satisfaction can be explained through this andragogical lens. Women with greater family responsibilities may perceive a higher “practical utility” in a digital skills program that offers tools for flexible employment or household management. As Kasworm (2003) notes, adult learners view the classroom as a bridge to their social world; for a mother heading a household, a program that facilitates labor market reintegration directly addresses her immediate “life-space” needs, thereby enhancing her perception of relevance and satisfaction.

### 2.4 Empowerment, autonomy, and social determinants

The framework also incorporates the sociological concept of economic empowerment. According to UN Women (2022), empowerment is a transformative process that strengthens women's capacity for autonomous decision-making. Continuing education acts as a strategic instrument for this transformation by reducing structural inequalities. However, the “conditions of entry” into education are not equal.

Sociodemographic factors—such as household headship and decision-making autonomy—act as social determinants of the learning experience. In this study, we distinguish between three dimensions of autonomy: family finances, personal finances, and personal time. While Boateng et al. (2022) emphasize that autonomy is a multifaceted construct, our findings suggest that these individual traits have a limited impact on the final evaluation of a program (*R*^2^ = 0.035). This leads to a critical sociological conclusion: while women's personal circumstances shape their initial approach to learning, the institutional and pedagogical quality of the program remains the primary driver of the educational experience.

### 2.5 Perceived service quality and the shift to institutional factors

Finally, the research draws on the SERVQUAL model by Parasuraman et al. (1988), which identifies five dimensions of service quality: reliability, responsiveness, assurance, empathy, and tangibles. This theory suggests that student satisfaction is fundamentally determined by the “gap” between what the institution promises and what it delivers in these dimensions.

The low explanatory power of our individual-level model indicates that the “missing variance” in satisfaction likely resides in these institutional and pedagogical factors. As Dhawan (2022) and Tsinidou et al. (2010) have demonstrated, teaching quality, curriculum relevance, and administrative support are consistently the strongest predictors of satisfaction. Therefore, this framework shifts the focus from “who the participants are” to “how the program is delivered,” arguing that high-quality, flexible, and supportive educational designs can achieve high satisfaction across diverse socioeconomic profiles.

## 3 Methodology

### 3.1 Study context

The empirical setting of this study is the Diploma in Digital Competencies for Employment, a university continuing education program specifically designed to strengthen the employability and economic autonomy of adult women in Chile. The diploma was developed and implemented by Universidad Alberto Hurtado as part of a broader public initiative that sought to expand women's labor market opportunities through lifelong learning and digital skills development.

The program formed part of the project *Transfer, Training, and Promotion of Employment for Women in Urbanized Territories of the Metropolitan Region of Santiago*, funded by the Regional Government of Santiago in response to the socioeconomic consequences of the COVID-19 pandemic. The initiative aimed to reduce structural barriers that frequently limit women's labor market participation, including caregiving responsibilities, digital exclusion, and restricted access to formal education and vocational training. The project was officially approved by the Metropolitan Regional Council under Act No. 270-22 on June 22, 2022.

The intervention was implemented across the 34 municipalities of Greater Santiago and was inspired by the *Mujeres Emplea* initiative promoted jointly by the International Labour Organization (ILO), UN Women, the Economic Commission for Latin America and the Caribbean (ECLAC), the Food and Agriculture Organization of the United Nations (FAO), and the Office of the United Nations Resident Coordinator. Although the outcomes of *Mujeres Emplea* have not been comprehensively systematized, the initiative highlighted the importance of integrating employability training with actions that strengthen women's decision-making autonomy, digital competencies, and economic empowerment.

Within this framework, Universidad Alberto Hurtado designed and delivered a university-certified diploma that combined technical digital skills with broader competencies intended to facilitate labor market reintegration and strengthen participants' confidence, employability, and economic autonomy. The curriculum was specifically tailored to the needs of adult women with diverse educational backgrounds and family responsibilities, offering flexible training opportunities that supported both professional development and social inclusion.

Because the program targeted women facing heterogeneous socioeconomic and family circumstances, it provides an appropriate setting for examining whether individual characteristics—including educational attainment, household responsibilities, and decision-making autonomy—are associated with participants' overall satisfaction with the educational experience. Consequently, this intervention offers an informative case for understanding the extent to which participant characteristics contribute to explaining satisfaction in publicly funded continuing education programs aimed at promoting women's economic empowerment.

### 3.2 Study design

This study adopted a quantitative, cross-sectional, and correlational research design to examine the association between selected individual characteristics and participants' overall satisfaction with a university continuing education program. The study did not seek to establish causal relationships but rather to estimate the magnitude and direction of the associations between sociodemographic characteristics, decision-making autonomy, and satisfaction.

A cross-sectional design was considered appropriate because all information was collected at a single point in time following participants' completion of the diploma program. The correlational approach allowed the simultaneous examination of multiple predictors using multivariate statistical techniques while controlling for the associations of the remaining variables included in the model.

### 3.3 Participants

The analytical sample comprised 1,794 adult women who voluntarily completed a structured follow-up questionnaire upon program completion. This group represents 27.6% of all eligible graduates; thus, it should be treated as a self-selected subgroup rather than a strictly representative sample of the entire participant population. While this self-selection requires caution when generalizing the findings, the sample size remains robust for multivariate analysis, offering high statistical power. The participants exhibited significant diversity, with ages ranging from 18 to 75 years (M = 40.65, SD = 10) and a wide spectrum of socioeconomic backgrounds, including variations in household headship and income levels.

### 3.4 Measures

#### 3.4.1 Dependent variable

Program satisfaction was operationalized as a composite construct based on four items measured on a five-point Likert scale ranging from 1 (“Strongly disagree”) to 5 (“Strongly agree”). The items assessed participants' overall evaluation of the diploma, including perceived fulfillment of expectations, achievement of learning objectives, willingness to recommend the program, and perceived usefulness of the program's employment platform.

The four items were averaged to create a single satisfaction index. The scale demonstrated high internal consistency, with a Cronbach's alpha of 0.864 and a McDonald's omega coefficient of 0.868. Across the analytical sample, the composite satisfaction score presented a mean of 4.44 (SD = 0.71).

#### 3.4.2 Independent variables

The explanatory variables included participants' educational attainment, number of children, household headship, and decision-making autonomy.

Educational attainment was measured as an ordinal variable representing participants' highest completed educational level. Number of children was recorded as a continuous count variable.

Decision-making autonomy was assessed through three dimensions: autonomy over family finances, autonomy over personal finances, and autonomy over personal time. Each dimension was measured using a four-category scale ranging from 1 (“Another family member decides without my participation”) to 4 (“I decide mainly”).

Household headship was included as a dichotomous variable, coded as 1 = not household head and 2 = household head. Approximately two-thirds of participants identified themselves as household heads.

#### 3.4.3 Income variables

Personal monthly income and household monthly income were initially considered as potential control variables because they could reflect participants' socioeconomic conditions. Preliminary regression analyses indicated that neither variable was significantly associated with program satisfaction, nor did their inclusion improve the explanatory power of the model or alter the estimated effects of the substantive predictors. In addition, both variables exhibited highly skewed distributions with several extreme values, reflecting the considerable socioeconomic heterogeneity of the participants. Because a substantial proportion of respondents were unemployed or reported no personal income, self-reported income was considered to have limited conceptual value for explaining satisfaction in this context. Consequently, both income variables were excluded from the final regression model, although their descriptive statistics are reported to characterize the sample.

### 3.5 Statistical analysis

All statistical analyses were conducted using IBM SPSS Statistics Version 26 (IBM Corp., Armonk, NY, USA).

The analytical strategy consisted of four sequential stages. First, descriptive statistics were calculated to summarize participants' characteristics and the distribution of the study variables. Second, the internal consistency of the satisfaction scale was assessed using Cronbach's alpha and McDonald's omega coefficients. Third, Pearson correlation coefficients were computed to examine the bivariate associations among the variables included in the study. Finally, multiple linear regression analysis was performed to estimate the independent contribution of each predictor to overall program satisfaction while controlling for the remaining explanatory variables.

Before estimating the regression model, the assumptions underlying multiple linear regression were evaluated. Particular attention was given to multicollinearity through tolerance values and Variance Inflation Factors (VIF). All VIF values remained below 2, indicating the absence of problematic multicollinearity and supporting the stability of the estimated regression coefficients.

Although descriptive information on personal and household income was retained to characterize the socioeconomic diversity of the participants, these variables were excluded from the final regression model because they were neither statistically significant nor theoretically informative in explaining program satisfaction. This decision resulted in a more parsimonious model focused on variables with greater conceptual relevance to the objectives of the study.

## 4 Results

The primary objective of the analysis was to examine participants' satisfaction with the diploma-format learning program and to identify individual variables that may be statistically related to this satisfaction as the dependent variable. The analytical sample comprised 1,794 adult women who completed the follow-up questionnaire.

### 4.1 Descriptive analysis and reliability

Findings reveal high levels of agreement regarding the program's value. On a 5-point Likert scale, the perception that the program provided the expected tools and knowledge reached a mean of 4.31 (SD = 0.878), and the belief that the diploma would enable the achievement of initial objectives yielded a mean of 4.41 (SD = 0.835). The willingness to recommend the diploma to other women presented a mean of 4.68 (SD = 0.739), while the associated employment portal was evaluated with a mean of 4.37 (SD = 0.893).

The composite measure of general satisfaction, constructed as the average of four evaluation items, yielded a mean of 4.44 (SD = 0.71). The scale demonstrated high internal consistency, with a Cronbach's alpha of 0.864 and a McDonald's omega of 0.868. The observed range encompassed the full scale (1–5), with a skewness of −1.781 and a kurtosis of 4.070. Table 1 reports the descriptive statistics for the key variables included in the model.

**Table 1:** Descriptive statistics of key variables.

Code	Variable	Mean	Standard deviation
1	Program satisfaction	4.44	0.71
2	Number of children	1.37	1.18
3	Autonomy over family finances	2.22	0.82
4	Autonomy over personal finances	2.81	0.50
5	Autonomy over personal time	2.80	0.49
6	Household headship	1.66	0.47
7	Highest educational attainment	2.27	0.87

The high mean satisfaction score (M = 4.44) indicates a consistently positive evaluation across diverse groups. Variables such as the number of children and household headship reflect the heterogeneous care burdens and economic responsibilities that define the participants' social reality. The autonomy measures, clustering around mid-scale values, suggest a context of partial decision-making power, highlighting the ongoing negotiation of gendered roles and household authority prior to the intervention.

### 4.2 Correlation analysis

Table 2 presents the Pearson correlation coefficients among the study variables. Program satisfaction showed weak but significant correlations with number of children (*r* = 0.109, *p* < 0.01) and household headship (*r* = 0.064, *p* < 0.01), while educational attainment was negatively associated with satisfaction (*r* = −0.158, *p* < 0.01). No significant correlations were observed between satisfaction and autonomy indicators.

**Table 2:** Pearson correlations between variables.

Code	Variable	1	2	3	4	5	6	7
1	Program satisfaction	1.000	0.109^**^	0.007	−0.035	0.031	0.064^**^	−0.158^**^
2	Number of children	0.109^**^	1.000	0.225^**^	−0.038	0.001	0.195^**^	−0.276^**^
3	Autonomy over family finances	0.007	0.225^**^	1.000	0.294^**^	0.267^**^	0.596^**^	−0.115^**^
4	Autonomy over personal finances	−0.035	−0.038	0.294^**^	1.000	0.348^**^	0.178^**^	0.016
5	Autonomy over personal time	0.031	0.001	0.267^**^	0.348^**^	1.000	0.172^**^	−0.067^**^
6	Household headship	0.064^**^	0.195^**^	0.596^**^	0.178^**^	0.172^**^	1.000	−0.155^**^
7	Educational attainment	−0.158^**^	−0.276^**^	−0.115^**^	0.016	−0.067^**^	−0.155^**^	1.000

Significant bivariate associations between satisfaction and the number of children or household headship suggest that women facing higher domestic demands may perceive greater instrumental utility in the program. Conversely, the negative correlation with educational attainment points to prior academic trajectories acting as a critical filter for quality expectations. The weak magnitude of these correlations overall indicates that individual traits and autonomy dimensions alone provide only a limited explanation for the variance in satisfaction, suggesting that the educational experience may transcend these entry conditions. ^**^ indicates statistical significance at the 0.01 level (two-tailed).

The autonomy dimensions showed significant but moderate associations, particularly between autonomy over family finances and autonomy over personal time (*r* = 0.267, *p* < 0.01). Household headship showed the strongest association with autonomy over family finances (*r* = 0.596, *p* < 0.01). Correlation coefficients remained below critical levels, suggesting that multicollinearity was unlikely to affect subsequent regression analyses.

### 4.3 Multiple regression analysis

A multiple linear regression analysis was conducted to examine whether individual characteristics and autonomy-related variables predicted program satisfaction after controlling for the simultaneous effect of all predictors. The dependent variable was overall program satisfaction, and the predictors included educational attainment, autonomy over personal finances, household headship, number of children, autonomy over personal time, and autonomy over family finances.

#### 4.3.1 Model fit

Table 3 presents the summary statistics of the multiple regression model. The model showed a statistically significant association between the set of predictors and program satisfaction, *F*_(6, 1, 787)_ = 10.802, *p* < 0.001. The correlation between observed and predicted values was weak (*R* = 0.187), and the model explained 3.5% of the variance in program satisfaction (*R*^2^ = 0.035; Adjusted *R*^2^ = 0.032).

**Table 3:** Model summary.

Model	*R*	*R^2^*	Adjusted *R^2^*	Std. error of estimate	Δ*R^2^*	Δ*F*	*df1*	*df2*	Sig. ΔF	Durbin-Watson
1	0.187	0.035	0.032	0.695	0.035	10.802	6	1,787	< 0.001	2.041

Source: SPSS output. Predictors: Educational attainment, autonomy over personal finances, household headship, number of children, autonomy over personal time, autonomy over family finances. Dependent variable: Program satisfaction.

The standard error of the estimate was 0.695, indicating the average deviation between observed and predicted satisfaction scores. The Durbin–Watson statistic was 2.041, suggesting that residual independence assumptions were adequately satisfied.

Table 4 presents the results of the multiple linear regression model examining the associations between sociodemographic characteristics, decision-making autonomy, and overall satisfaction with the diploma program. The model was statistically significant, although it explained a relatively small proportion of the variance in satisfaction (*R*^2^ = 0.035).

**Table 4:** ANOVA results.

Model	Sum of squares	*df*	Mean square	*F*	Sig.
Regression	31.345	6	5.224	10.802	< 0.001
Residual	864.265	1,787	0.484		
Total	895.610	1,793			

Source: SPSS output. Dependent variable: Program satisfaction.

#### 4.3.2 Regression coefficients

Table 5 presents the estimated coefficients of the multiple regression model. The coefficients indicate the individual statistical contribution of each predictor after controlling for the remaining variables.

**Table 5:** Regression coefficients.

Model	Unstandardized coefficients		Standardized coefficients	*t*	Sig.	95,0% confidence interval for B		Correlations		Collinearity statistics		
	B	Std. error	Beta			Lower limit	Upper limit	Zero order	Partial	Part	Tolerance	VIF
Constant	4,565	0.135		33,867	0.000	4,301	4,829					
Number of children	0.043	0.015	0.072	2,902	0.004	0.014	0.073	0.109	0.068	0.067	0.872	1,147
Autonomy over family finances	−0.056	0.026	−0.065	−2,140	0.033	−0.107	−0.005	0.007	−0.051	−0.050	0.581	1,722
Autonomy over personal finances	−0.053	0.036	−0.037	−1,456	0.146	−0.123	0.018	−0.035	−0.034	−0.034	0.826	1,211
Autonomy over personal time	0.059	0.036	0.041	1,618	0.106	−0.012	0.130	0.031	0.038	0.038	0.844	1,184
Household headship	0.101	0.043	0.068	2,327	0.020	0.016	0.186	0.064	0.055	0.054	0.636	1,573
Educational attainment	−0.107	0.020	−0.131	−5,384	0.000	−0.146	−0.068	−0.158	−0.126	−0.125	0.908	1,101

Source: While several individual predictors achieve statistical significance, their small effect sizes and the low overall explanatory power (R^2^ = 0.035) require caution in interpretation. These results reveal a positive evaluation of the program within existing gendered contexts—particularly for those with greater family responsibilities—but they do not reveal an elimination of structural inequalities or caregiving burdens. The stability of the coefficients (VIF < 2) confirms that satisfaction is a complex, multicausal phenomenon where pedagogical and institutional factors likely exert a more decisive influence than sociodemographic profiles.

Educational attainment showed the strongest standardized association with program satisfaction and was negatively related to satisfaction (*B* = −0.107, *β* = −0.131, *p* < 0.001). This indicates that higher educational attainment was associated with slightly lower satisfaction scores when the other predictors were held constant.

The number of children showed a positive and statistically significant association with satisfaction (*B* = 0.043, *β* = 0.072, *p* = 0.004). Household headship also presented a positive association with satisfaction (*B* = 0.101, *β* = 0.068, *p* = 0.020).

Autonomy over family finances was negatively associated with satisfaction (*B* = −0.056, *β* = −0.065, *p* = 0.033). In contrast, autonomy over personal finances (*B* = −0.053, *β* = −0.037, *p* = 0.146) and autonomy over personal time (*B* = 0.059, *β* = 0.041, *p* = 0.106) were not statistically significant predictors in the multivariate model.

Overall, the standardized coefficients indicated small effect sizes, with the largest association observed for educational attainment (*β* = −0.131).

#### 4.3.3 Regression diagnostics

The collinearity diagnostics indicated acceptable levels of predictor independence. Variance Inflation Factor (VIF) values ranged from 1.101 to 1.722, while tolerance values ranged from 0.581 to 0.908. These values remained below commonly accepted thresholds, indicating that multicollinearity did not represent a concern in the regression model.

Although some predictors showed moderate correlations in the preliminary analysis, particularly household headship and autonomy over family finances (*r* = 0.596), these associations did not compromise the stability of the regression estimates.

## 5 Discussion

### 5.1 Main findings: high satisfaction and limited predictive capacity

The findings of this study reveal a very high level of satisfaction among participants enrolled in the *Diploma in Digital Skills for Employment*, with an overall mean satisfaction score of 4.44 (SD = 0.71). This result indicates that participants evaluated the program positively in terms of its perceived value, usefulness, and contribution to their initial objectives. The consistency of these evaluations suggests that the diploma successfully addressed important expectations among adult learners.

However, the central contribution of this study lies in the contrast between the high level of satisfaction observed and the limited explanatory capacity of the predictive model. Although the regression model was statistically significant, the individual-level variables included in the analysis explained only 3.5% of the variance in satisfaction (*R*^2^ = 0.035). This finding suggests that satisfaction in adult education contexts is a complex phenomenon that cannot be adequately explained through sociodemographic characteristics and autonomy-related factors alone.

The distinction between statistical significance and substantive relevance is particularly important in interpreting these findings. Given the large sample size (*n* = 1,794), the analysis had sufficient statistical power to detect relatively small associations. Nevertheless, the standardized coefficients were consistently modest, indicating that the predictors examined have limited practical influence on satisfaction evaluations. Therefore, while individual characteristics may contribute to differences in satisfaction, they appear to represent only a small component of the overall learning experience.

This result is consistent with previous research emphasizing that educational satisfaction is influenced by multiple dimensions, including instructional quality, curriculum relevance, interaction with instructors, institutional support, and perceived learning outcomes (Tinto, 1975; Elliott and Shin, 2002). Consequently, future explanatory models should incorporate program-level, motivational, and experiential variables to better capture the mechanisms underlying satisfaction among adult learners.

From a sociological perspective, it is essential to clarify what this high satisfaction reveals—and what it does not. These findings indicate that the program is perceived as a valuable resource for labor market navigation, particularly by women facing significant care burdens and household responsibilities. However, this satisfaction should not be interpreted as evidence of the elimination of structural gender inequalities or the reduction of domestic demands. Instead, the higher satisfaction among women with more children or those heading households reveals an instrumental evaluation situated within a context of “time poverty” and economic pressure. In this sense, satisfaction acts as a measure of the program's functional relevance to the participants' immediate social reality rather than a marker of transformed autonomy or a change in the underlying gendered barriers to continuing education.

### 5.2 Educational attainment and expectation-disconfirmation

Among the predictors included in the model, educational attainment showed the strongest standardized association with program satisfaction and was negatively related to satisfaction (*B* = −0.107, *β* = −0.131, *p* < 0.001). This finding indicates that participants with higher educational attainment tended to report slightly lower satisfaction levels after controlling for other characteristics.

One possible explanation for this relationship can be found in Expectation-Disconfirmation Theory (Oliver, 1980), which proposes that satisfaction emerges from the comparison between initial expectations and perceived performance. Individuals with higher educational backgrounds may enter training programs with more developed evaluative frameworks and higher expectations regarding academic rigor, content depth, and pedagogical quality. Consequently, when a program is designed for participants with heterogeneous levels of previous knowledge, those with greater educational experience may perceive smaller improvements or fewer benefits relative to their initial expectations.

This interpretation is also consistent with broader perspectives on service quality and satisfaction. Parasuraman et al. (1988) argue that evaluations of services depend not only on objective performance but also on the alignment between expectations and perceived outcomes. Similarly, Bhattacherjee (2001) highlights the role of previous experiences and expectations in shaping users' evaluations of services. In educational settings, these mechanisms may explain why participants with stronger academic backgrounds apply more demanding criteria when evaluating learning experiences.

Nevertheless, this interpretation should be considered cautiously because the study did not directly measure participants' expectations before enrollment, perceived learning gains, or changes in knowledge. Future research should examine whether expectation levels mediate the relationship between educational background and satisfaction.

### 5.3 Family responsibilities and instrumental motivations

The positive associations between number of children and satisfaction (*B* = 0.043, *β* = 0.072, *p* = 0.004) and between household headship and satisfaction (*B* = 0.101, *β* = 0.068, *p* = 0.020), although small in magnitude, provide relevant insights into how adult learners may evaluate educational opportunities according to their personal circumstances.

From the perspective of adult learning theory, adults are more likely to value educational experiences when they perceive direct connections between learning and their immediate needs, responsibilities, and life contexts (Knowles, 1973). Similarly, research on adult learners emphasizes the importance of instrumental motivations, particularly when education is perceived as a resource for addressing practical challenges or improving personal and professional opportunities (Kasworm, 2003).

For women with greater family responsibilities, the acquisition of digital competencies may represent a meaningful resource associated with employment opportunities, increased confidence, improved access to information, or better management of daily activities. Therefore, these participants may perceive greater practical utility from the program, contributing to slightly higher satisfaction evaluations.

However, these interpretations remain exploratory. The current study did not directly assess participants' motivations, employment expectations, caregiving responsibilities, or perceived usefulness of acquired skills. Future research should investigate whether these factors mediate the relationship between family characteristics and satisfaction.

### 5.4 Implications for adult education programs

The findings have important implications for the design and implementation of adult education initiatives. First, the limited explanatory power of individual-level predictors suggests that satisfaction should not be primarily understood as a consequence of participants' demographic profiles. Instead, greater attention should be directed toward educational processes and institutional conditions that shape the learning experience.

Programs targeting adult learners should consider strategies such as differentiated learning pathways, flexible delivery formats, curriculum adaptation, and stronger alignment between content difficulty and participants' previous educational experiences. In particular, the lower satisfaction observed among participants with higher educational attainment suggests the importance of offering more advanced or specialized learning opportunities for individuals who already possess greater academic backgrounds.

At the institutional level, the findings highlight the need to evaluate satisfaction through a multidimensional perspective that includes not only learner characteristics but also pedagogical and contextual factors. Improving instructional quality, strengthening instructor support, and ensuring the practical relevance of course content may represent more effective strategies for enhancing satisfaction than focusing exclusively on participant characteristics.

## 6 Limitations

Although this study provides relevant empirical evidence based on a large sample of adult learners, several limitations should be acknowledged when interpreting the findings.

### 6.1 Sample selection and self-selection bias

The study relied on a voluntary follow-up questionnaire with a response rate of 27.6%. Sociologically, this introduces a risk of self-selection bias: participants who responded may represent women who successfully overcame structural barriers and care burdens, potentially masking the experiences of those with higher “time poverty” or less domestic support [i, 87]. Therefore, caution is warranted when generalizing these findings to the entire population of adult learners.

### 6.2 Distributional constraints: the ceiling effect

A significant statistical limitation is the pronounced “ceiling effect” in the satisfaction scores (Skewness = −1.781). This high concentration of positive evaluations limited the model's variability and its ability to detect more complex relationships between gendered inequalities and satisfaction. Future research should use qualitative instruments to capture more nuanced differences in experiences that quantitative scales might miss.

### 6.3 Cross-sectional design and causal limitations

The cross-sectional nature of the design allows for identifying associations but precludes establishing causal relationships. For instance, it remains unclear whether high satisfaction is a result of prior autonomy or if the program itself enhanced participants' perceived agency. This distinction is crucial for understanding whether educational interventions effectively reduce gendered barriers or simply attract women who already possess higher levels of autonomy [i, 158].

### 6.4 Limited scope of the predictive model

The low explanatory power () indicates that the sociodemographic and autonomy variables captured only a small fraction of what shapes satisfaction. This reveals that satisfaction is not primarily a consequence of participants' demographic profiles. Important determinants—such as the specific intensity of care burdens, instructional quality, and tutor interaction—were not directly measured. These programmatic and contextual factors likely play a more decisive role in the educational experience of adult women.

## 7 Conclusions

The findings of this study demonstrate that participants reported very high levels of satisfaction with the diploma-format learning program. Evaluations of perceived quality, practical usefulness, achievement of objectives, and willingness to recommend the program were consistently positive, and the satisfaction scale showed high internal consistency. These results indicate that participants perceived the initiative as a valuable educational experience within the context of adult learning and continuing education.

However, the main contribution of this study lies in demonstrating that high satisfaction does not necessarily translate into strong explanatory models based on individual characteristics. Although educational attainment, number of children, household headship, and autonomy over family finances showed statistically significant associations with satisfaction, their effects were small. The regression model explained only a limited proportion of variance, suggesting that sociodemographic and autonomy-related factors provide only a partial understanding of satisfaction differences among participants.

These findings highlight the multidimensional nature of satisfaction in adult education. While participant characteristics may influence how learning experiences are evaluated, broader contextual and program-related factors are likely to play a more decisive role. Variables such as instructional quality, curriculum relevance, instructor support, perceived usefulness, and alignment between program content and learner expectations should therefore be incorporated into future explanatory models.

From a practical perspective, the results suggest that continuing education initiatives for adult women should prioritize aspects directly related to the learning experience, including flexible delivery formats, differentiated learning pathways, effective pedagogical support, and content adaptation according to participants' previous educational backgrounds and needs. In particular, the lower satisfaction observed among participants with higher educational attainment suggests the importance of designing advanced or differentiated learning opportunities for heterogeneous groups of adult learners.

Overall, this study contributes to the literature on adult education by showing that satisfaction with training programs is not primarily determined by participants' individual profiles but emerges from the interaction between learner characteristics and the educational environment. Future research should adopt more comprehensive approaches, integrating individual, motivational, and institutional dimensions to better understand how adult learning experiences generate satisfaction and perceived value.

## Data Availability

The original contributions presented in the study are included in the article/supplementary material, further inquiries can be directed to the corresponding author.
