# Correction: Associations between individual characteristics, decision-making autonomy, and satisfaction in a continuing education program for adult women in Chile

**DOI:** 10.3389/fsoc.2026.2013118

**Published:** 2026-10-16

**Authors:** David Álvarez-Maldonado, Javier Labbé Cid, Nicolás Pablo Barrientos Oradini, Carmen Pénnanen Arias, Luis Felipe Vergara, Víctor Manuel Yáñez Jara, Rafael Romero-Meza, Juan Carlos Armijos

**Affiliations:** 1 Universidad Tecnológica Metropolitana, Santiago, Chile; 2 Instituto de Salud Pública, Universidad Andrés Bello, Santiago, Chile; 3 Faculty of Economics and Business, Universidad Alberto Hurtado, Santiago, Chile; 4 School of Business and Administration, Universidad Miguel de Cervantes, Santiago, Chile; 5 Investigadora independiente, Santiago, Chile; 6 Faculty of Business and Economy, Universidad Andrés Bello, Santiago, Chile; 7 Academic and Administrative Area, Institute of Public Health, Universidad Andrés Bello, Santiago, Chile; 8 Universidad Alberto Hurtado, Santiago, Chile; 9 Facultad de Economía y Negocios, Universidad Santo Tomás, Santiago, Región Metropolitana, Chile; 10 School of Business and Administration, Faculty of Engineering, Science and Technology, Bernardo O'Higgins University, Santiago, Chile

**Keywords:** academic satisfaction, adult education, autonomy, multiple regression, sociodemographic factors

Affiliation 11 Faculty of Business and Technology, University of Arts, Sciences and Communication (UNIACC), Santiago, Chile was erroneously included in the paper. This affiliation has now been removed for author(s) Juan Carlos Armijos.

The original version of this article has been updated.

